# Microwave-Accelerated McKenna Synthesis of Phosphonic Acids: An Investigation

**DOI:** 10.3390/molecules28083497

**Published:** 2023-04-15

**Authors:** Dana Mustafa, Justin M. Overhulse, Boris A. Kashemirov, Charles E. McKenna

**Affiliations:** Department of Chemistry, University of Southern California, Los Angeles, CA 90089, USA

**Keywords:** microwave-assisted synthesis, phosphonic acids, bromotrimethylsilane, dealkylation

## Abstract

Phosphonic acids represent one of the most important categories of organophosphorus compounds, with myriad examples found in chemical biology, medicine, materials, and other domains. Phosphonic acids are rapidly and conveniently prepared from their simple dialkyl esters by silyldealkylation with bromotrimethylsilane (BTMS), followed by desilylation upon contact with water or methanol. Introduced originally by McKenna, the BTMS route to phosphonic acids has long been a favored method due to its convenience, high yields, very mild conditions, and chemoselectivity. We systematically investigated microwave irradiation as a means to accelerate the BTMS silyldealkylations (MW-BTMS) of a series of dialkyl methylphosphonates with respect to solvent polarity (ACN, dioxane, neat BTMS, DMF, and sulfolane), alkyl group (Me, Et, and iPr), electron-withdrawing P-substitution, and phosphonate–carboxylate triester chemoselectivity. Control reactions were performed using conventional heating. We also applied MW-BTMS to the preparation of three acyclic nucleoside phosphonates (ANPs, an important class of antiviral and anticancer drugs), which were reported to undergo partial nucleoside degradation under MW hydrolysis with HCl at 130–140 °C (MW-HCl, a proposed alternative to BTMS). In all cases, MW-BTMS dramatically accelerated quantitative silyldealkylation compared to BTMS with conventional heating and was highly chemoselective, confirming it to be an important enhancement of the conventional BTMS method with significant advantages over the MW-HCl method.

## 1. Introduction

Phosphonic acids constitute one of the most important categories of organophosphorus compounds. The diverse utility of phosphonates encompasses applications as antibiotic and antiviral agents, chelating agents in imaging, catalysts, ion exchange materials, and components of polymers used in paints and adhesives, among many others [1]. As a result, efficient preparative routes to phosphonic acid derivatives are a subject of continuing interest in synthetic chemistry [2,3,4].

When present as part of a complex molecule, phosphonates are normally maintained as simple alkyl esters until final conversion to the final phosphonic acid product. Since its introduction by the senior author [5,6,7], silyldealkylation by bromotrimethylsilane (BTMS) became established as a standard method for accomplishing this transformation [1,8] as the resulting bis(trimethylsilyl) phosphonate is facilely desilylated upon contact with water or methanol. BTMS silyldealkylation is compatible with a wide range of acid-, base-, and hydrogenation-sensitive functional groups, including alkenyl, alkynyl, alkoxyalkyl, benzyl, benzoyl, diazomethyl, carboxamide, and carboxylate ester groups [3,7,8].

Under typical reaction conditions, BTMS silyldealkylations of phosphonate dialkyl esters proceed at room temperature or with moderate thermal heating. A growing trend in organic synthesis during recent years has been the substitution of microwave (MW) irradiation for conventional heating, with organophosphorus chemistry being no exception [9]. Some years ago, Kumar briefly examined the effect of microwave heating on the reaction of BTMS in acetonitrile (ACN) with a series of ethyl phosphonates, phosphoramides, and phosphates [10]. Yields > 95% (NMR) were achieved within a few minutes, whereas conventional heating required up to several hours to complete the reactions. These microwave-accelerated BTMS de-ethylation reactions were tolerant of bromoalkyl, piperazine, and nitrile moieties. The successful application of these conditions in preparing the (sulfonyl)methyl phosphonic acid analogues of prenyl diphosphates was subsequently reported by Tantillo [11]. Meziane [12] examined the microwave acceleration of diethyl phosphonate silyldealkylation by the Morita reagent (chlorotrimethylsilane and sodium iodide), which gave inferior yields compared to those obtained with BTMS and was less convenient in the workup phase. More recently, incidental examples of MW-assisted BTMS reactions with a dialkyl phosphonate ester have been reported by Maguire [13] and ourselves [14].

**Figure 1:**
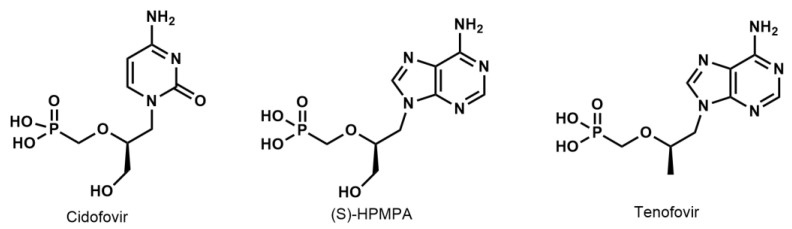
Examples of acyclic nucleoside phosphonates (ANPs) with antiviral activity.

In 2012, Jansa et al. studied the acid-catalyzed hydrolysis of acyclic nucleoside phosphonate (ANP) esters under microwave irradiation [15]. ANPs are a group of nucleotide analogues that possess a broad spectrum of biological activity and include an array of important antiviral agents (Figure 1) [16,17]. Using 2–3 molar equivalents of aqueous HCl and temperatures of 130–140 °C in a sealed vessel, ethyl and isopropyl phosphonate esters were hydrolyzed in 20–30 min. The hydrolysis mechanism was suggested to involve a carbocation intermediate, consistent with a reactivity order: iPr esters > Et esters. However, in the case of diaminopurine (DAP) nucleoside phosphonates, partial hydrolysis of the C6 amino group was observed, leading to the formation of 10% guanine side products. This side reaction was reduced to 5% by lowering the temperature to 130 °C and using a lower concentration of HCl. As such, yields of products varied between 77% and 93% [15]. The authors claimed that this method has the advantage of being a ‘green’ alternative, an assertion subject to challenge [2]. In place of HCl for the hydrolysis of several phosphinate esters, the latter authors investigated the use of a catalytic amount of p-toluenesulfonic acid (PTSA), which was shown to be an effective substitute, albeit still requiring high reaction temperatures (160–180 °C), sealed reaction vessels, and reaction times of 0.5–5 h [2].

Here, we present a systematic investigation of the scope of the microwave-assisted BTMS (MW-BTMS) dealkylation of phosphonate alkyl esters, comparing the influence of the alkyl (methyl, ethyl, or isopropyl) ester groups on the 100% conversion time and examining the role of the solvent. The effect of an electron-withdrawing P-substituent is examined, as is the chemoselectivity for trialkyl phosphonoacetates. Finally, we assess its compatibility with three ANP phosphonate esters possessing acid-sensitive groups: PMEDAP (OiPr)_2_, (S)-PMPDAP (OEt)_2_, and PMEA (OiPr)_2_ [10]. MW-BTMS (and thermally heated BTMS) reactions were typically performed at 40 or 60 °C with a small molar excess of BTMS. In a few cases, higher temperatures were used (80 or 110 °C), but these never exceeded the standard boiling point of the solvent. All reactions were carried out at ambient pressure and protected from moisture with a drying tube.

## 2. Results

**Scheme 1:**
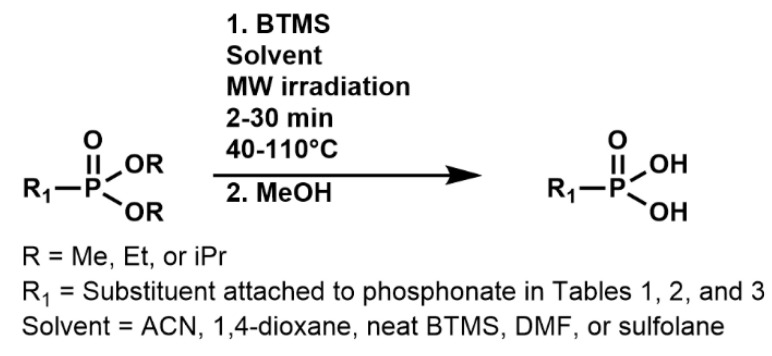
General reaction scheme for microwave-assisted BTMS dealkylation in the conversion of phosphonate alkyl esters to phosphonic acids.

Scheme 1 outlines the general reaction scheme adopted to investigate the effects of reaction conditions on microwave-assisted BTMS phosphonate ester dealkylations. Dimethyl-, diethyl-, or diisopropyl methylphosphonate reacted with 2 equiv of BTMS in a commercial thermostat microwave oven. Solvent and temperature were varied to study their effect on reaction times and yields, which are listed in Table 1 together with the corresponding results for control reactions performed under the same reaction conditions, except for the replacement of microwave irradiation by conventional heating with a sand bath. The time to the completion of silyldealkylation with MW-BTMS was determined by ^31^P NMR, and after treatment with MeOH, the yield of isolated methylphosphonic acid was obtained.

Reaction completion times were compared for four aprotic solvents with markedly different structures, with sulfolane being the most polar (ε = 43), followed by ACN (ε = 37), DMF (ε = 38), and 1,4-dioxane, which is the least polar (ε = 2.2) [18]. As a reference, reactions in neat BTMS were also run [5]. The results are presented in Table 1. As the enhanced rate of microwave-assisted reactions involves the excitation of polar bonds in molecules, solvent polarity is expected to influence the absorption of the radiant energy from the microwave source and thereby affect the reaction rate. 

For the methyl ester, shorter reaction times were observed in DMF and sulfolane compared to ACN when the temperature and other conditions were held constant, although the dielectric constant (ε) of DMF is marginally lower than that of ACN. Significantly, in 1,4-dioxane, the least polar of the solvents tested, this reaction proceeded to completion at least as rapidly as in ACN. Rapid conversions were also observed for the reactions in neat excess BTMS. For the ethyl ester, the reaction in all four solvents was complete at 40 °C within 6–15 min. The reaction of the isopropyl ester required only 2 min in sulfolane at 80 °C and 20–30 min in ACN, DMF, or 1,4-dioxane at 60–80 °C.

In all cases, MW-BTMS reactions were up to >100 times faster (often complete within a few min) than their conventionally heated counterparts (not always complete after several hours), depending on the ester alkyl group, temperature, and BTMS molar excess. The reaction of all three esters was complete in neat BTMS within 2–15 min at 40 °C.

The marked P-O vs. C-O chemoselectivity of BTMS for silyldealkylation in carboxylate-phosphonate mixed esters under standard conditions is well established [7,19,20]. To investigate the conservation of this synthetically useful property under MW-BTMS, two model mixed esters, trimethyl- and triethylphosphonoacetate (Table 2, Entries 1 and 2), were reacted with BTMS under microwave irradiation in ACN at 60 °C. The results, displayed in Table 2, reveal that the quantitative dealkylation of these compounds requires more time than their simple methylphosphonate diester counterparts. As previously noted, the initial attack of the P=O group at the silicon atom of BTMS displacing Br^−^ creates a positive charge at phosphorus, decreasing reactivity when R’ is an electron-withdrawing substituent such as a carboxyl or α-halo alkyl group [5,21]. This effect persists in MW-BTMS, as exemplified by the dealkylation of diethyl (bromodifluoro)phosphonate (Table 2, Entry 4), which required a twofold molar excess of BTMS and irradiation at 60°C for 1 h.

**Figure 2:**
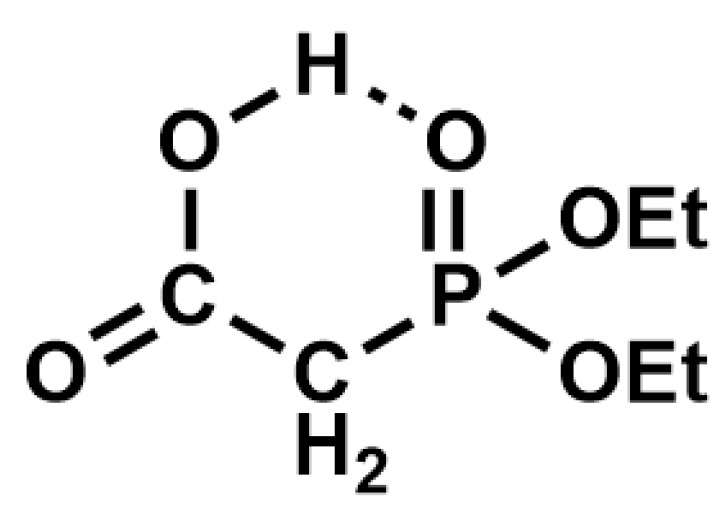
Internal hydrogen bonding in diethyl carboxyphosphonate decreases P=O oxygen nucleophilicity, leading to decreased silyldealkylation reactivity under MW-BTMS.

In contrast to the ethyl triester (Table 2, Entry 2), diethyl carboxylmethylphosphonate was appreciably less reactive under MW-BTMS in ACN, taking twice as long to complete with a threefold higher concentration of the BTMS reagent (Table 2, Entry 3). This may be due to the formation of an intramolecular hydrogen bond between the free carboxyl group and the P=O, which would decrease the nucleophilicity of the P=O oxygen in its rate-determining attack on silicon in BTMS (Figure 2).

Slight modifications to the workup procedures for the carboxylphosphonate products were necessary. For the silyl ester intermediate of 2-phosphonoacetic acid (Table 2, Entry 3), desilylation to the final product was performed with water because the use of methanol esterified the carboxylic acid group (for acid-sensitive compounds, hydrolysis with a neutral pH aqueous buffer is recommended) [22]. Importantly, for the mixed P-O and C-O triesters (Table 2, Entries 1 and 2), silylation is 100% selective at the P-O ester. However, the presence of trace amounts of unreacted BTMS produces HBr upon exposure to moisture, which can result in partial dealkylation at the carboxylate ester [19,20,23]. In order to avoid this, any excess BTMS must be removed under reduced pressure before the desilyation of the silyl ester intermediate. In addition to the deployment of a drying tube, the BTMS reactions with phosphonoacetates were carried out under dry nitrogen as an extra precaution against moisture.

ANPs, especially those containing DAP or PMEA groups, may undergo the partial degradation of the nucleoside moiety during phosphonate ester dealkylation under the vigorous HCl hydrolysis conditions described by Jansa et al. [15]. In contrast, the data presented in Table 3 show that the silyldealkylation of these compounds using MW-BTMS was quantitative within 15–30 min at 60 °C, depending on the molar ratio of BTMS to the ester substrate. Control reactions in which all reaction conditions were the same except for microwave irradiation (Table 3) revealed that the MW-BTMS dealkylations were completed 16–20 times faster.

## 3. Discussion

The initial report on the acceleration of BTMS silyldealkylations of certain diethyl phosphonates in ACN [11] was followed only sparsely by further examples [11,12,15], none of which defined the scope and comparative utility of this method. Recently, our laboratory demonstrated that under specific MW heating conditions for a BTMS dealkylation step, unwanted anomerization in the synthesis of dTTP analogues could be avoided [24]. Meanwhile, alternative MW-based approaches have been suggested [15] and criticized [2]. Here, we have systematically examined the effects of solvent polarity, alkyl ester structure, and temperature on MW-BTMS phosphonate diester silyldealkylation in comparison to thermal heating as a route to a series of phosphonic acids. Compared to conventional thermal heating of reaction mixtures, microwave irradiation can provide greatly shortened reaction times [10,25], cleaner reaction profiles [26], better reproducibility [27], and higher product yields [10,26]. 

Using BTMS reacting with a uniform dialkyl methylphosphonate substrate, MW irradiation under the experimental conditions (Materials and Methods) was found to accelerate reactions dramatically for all three types of alkyl ester in all four aprotic solvents and neat excess BTMS, with quantitative conversion by NMR obtained for the dimethyl ester in as little as 2 min at 40–60 °C in solvents with different polarities and neat BTMS (Table 1). Notably, the least polar solvent (1,4-dioxane) was as effective at 40 °C as ACN, which has an almost twentyfold larger dielectric constant. Microwave-assisted organic synthesis is believed to involve at least two different heating mechanisms: dipolar polarization and ionic conduction. Dipolar polarization transfers heat energy via the interaction of a reactant or solvent dipole with the applied oscillating electric field. Ionic conduction is caused by the oscillation of dissolved charged particles in the microwave field. Dielectric properties thus determine the heating characteristics of a particular material. Even if the solvent has a low dielectric constant, polar reactants will enable effective energy transfer [28]. We do not have ε values for the two key substrates, dimethyl methyl phosphonate and BTMS; however, as an approximation, we calculated their dipole moments (μ) as 3.11 and 3.92 D, respectively, using equilibrium geometry at ground state (Spartan ’20) and density functional models (ωB97X-D, 6-31G*), assuming a solvent environment of low polarity (ε = 3.7, similarly to ethyl ether). These values are similar to the μ for acetonitrile, 3.89 D. 

In ACN at 40 °C, the methyl ester reaction time was longer than for the other solvents (10 min vs. 2–5 min), but this was more than halved by raising the temperature to 60 °C. Results for the diethyl esters showed only a small decrease in reactivity, depending on the solvent. For the diethyl ester in 1,4-dioxane, the reaction time increased slightly to 15 min at 40 °C (vs. 7 h with conventional heating), but it was reduced to 6 min at 60 °C and 4 min at 80 °C. The reaction was virtually immediate in DMF, sulfolane, and ACN at 60 °C (2–5 min) and greatly accelerated compared to thermal heating alone. MW-BTMS silyldealkylation times of the sterically more hindered isopropyl diesters, which are typically less reactive with BTMS under conventional heating, did show a dependence on solvent polarity; sulfolane was the most effective (10 min at 60 °C and 2 min at 80 °C), compared to DMF (20 min at 80 °C, 5 min at 100 °C, and 2 min at 110 °C), 1,4-dioxane (30 min at 80 °C), and ACN (90 min at 40 °C and 30 min at 60 °C). The ability of MW-BTMS to achieve the rapid quantitative silylation of the isopropyl esters under these mild conditions is a signal improvement over the traditional BTMS method, where limitations to methyl or ethyl ester precursors of targeted phosphonic acids to avoid higher temperatures and longer reaction times may be encountered.

Neat BTMS reactions were used in the initially reported phosphonate diester silyldealkylations [5,6]. With simple dialkyl phosphonates, thermal heating reaction times in 5–70% excess BTMS ranged from less than an hour for a dimethyl ester to several hours for the corresponding diisopropyl ester. It was therefore of interest to assess the reaction performance of MW-BTMS carried out in the neat reagent. As shown in Table 1, quantitative silyldealkylation yields were achieved within 2 min from both the dimethyl and diethyl esters reacted at 40 °C, using 6 equiv of BTMS. Under the same conditions, the conversion of the diisopropyl ester was completed in 15 min.

A hallmark of the BTMS approach to the synthesis of phosphonic acids from their diesters is its chemoselectivity against the dealkylation of a carboxylic acid ester present in the same molecule [5]. Consequently, we investigated whether this selectivity is preserved under MW reaction conditions (Table 2). Both trimethyl and triethyl phosphonoacetate irradiated in ACN at 60 °C for 10 and 15 min, respectively, were cleanly converted to the corresponding C-methyl mono esters with 100% chemoselectivity, and C-alkyl phosphonic acids were isolated in 96–98% yield after desilylation of silyl ester intermediates. It was also possible to convert diethyl phosphonoacetic acid to the triacid in 99% isolated yield using a twofold excess of BTMS in ACN at 60 °C and a 30 min reaction time. The longer reaction times presumably reflect the effect of a relatively more electronegative 1-carbon substituent on the phosphonium-like transition state generated by the nucleophilic attack of the phosphoryl oxygen on the silicon atom in BTMS [6,21] (in addition to the proposed effect of internal H-bonding in the phosphonoacetic acid). We therefore repeated the reaction using diethyl bromodifluoromethylphosphonate (Table 2), a useful synthon for introducing the –CF_2_P(O)(OEt)_2_ moiety, which has an exceptionally electronegative phosphonate carbon. This compound required 1 h in ACN at 60 °C to complete silyldealkylation, suggesting that MW irradiation does not change the mechanism of the reaction in terms of it proceeding via an electron-deficient transition state.

BTMS silyldealkylation has often been a valuable tool for the preparation of biologically active phosphonic acids, including ANPs [3,4,11,13,14]. This is particularly the case when the pharmacophore in these compounds may be vulnerable to degradation under harsh hydrolysis conditions, an example of which is treatment with HCl at high temperatures [15]. We examined the applicability of MW-BTMS to three ANP alkyl esters that were reported to be sensitive to HCl hydrolysis [15]: PMEDAP(OiPr)_2_, (S)-PMPDAP(OEt)_2_, and PMEA(OiPr)_2_. The reaction times required for the quantitative conversion of these compounds to the bis(trimethylsilyl) esters by treatment with BTMS in ACN at 60 °C (conventional heating) ranged from 4 to 6 h (Table 3). In contrast, by applying MW-BTMS under otherwise identical conditions, the conversion was complete within 15–30 min. In neither case was significant decomposition detected (NMR), and pure phosphonic acid was obtained in an 82–91% yield after recrystallization.

With the ongoing interest in expanded methods to prepare phosphonic acids from diverse ester substrates [29,30], we believe that MW-BTMS is a suitable topic for further investigation as well as being a demonstrably valuable tool for practical synthesis. 

## 4. Materials and Methods

BTMS was purchased from Sigma-Aldrich (St. Louis, MO, USA) and distilled under nitrogen (greaseless glass joints) prior to use. Dimethyl methylphosphonate, trimethyl phosphonoacetate, and triethyl phosphonoacetate were purchased from Aldrich. Diethyl methylphosphonate was purchased from Alfa Aesar (now Thermo Scientific Chemicals, Ward Hill, MA, USA), while diisopropyl methylphosphonate was purchased from Lancaster Synthesis (Lancaster, UK). Diethyl (bromodifluoromethyl)phosphonate was purchased from Oakwood Products (Estill, SC, USA). Acetonitrile was purchased from EMD Chemicals (Port Wentworth, GA, USA) and distilled prior to use; Drisolv N,N-dimethylformamide was purchased from EMD Chemicals; 1,4-dioxane was purchased from Macron Chemicals (Avantor/VWR, Radnor, PA, USA) and distilled over sodium; sulfolane was purchased from Sigma-Aldrich and distilled under reduced pressure. Diethyl phosphonoacetate [31] and the ANP esters were synthesized according to published methods [32,33].

All glassware was oven-dried, solvents were AR reagent grade, and NMR spectra were obtained at 500 (^1^H) or 202 (^31^P) MHz on a Varian VNMRS-500 NMR. The microwave irradiation of reaction mixtures was carried out in a Milestone Ethos Synth Microwave Synthesis Labstation. The chemical shift references for ^1^H NMR spectra were the residual HDO (δ 4.79) in D_2_O or residual CHCl_3_ (δ 7.26) in CDCl_3_, while the ^31^P NMR spectra were referenced against an external 85% H_3_PO_4_ standard (δ 0.00). All chemical shift values (δ) are given in ppm, and NMR sample pH values were measured in 99.9% D_2_O without deuterium isotope corrections. The approximate concentration of the NMR samples was 1–3 mg/mL for isolated compounds. 

General procedure for the microwave-assisted dealkylation of methylphosphonate dialkyl esters (Table 1): the appropriate dialkyl methylphosphonate ester (1.8 mmol) in a 10 mL round bottom flask equipped with a reflux condenser and protected from moisture by a Drierite-filled tube was dissolved in 1 mL of solvent, and BTMS (3.8 mmol) was rapidly added dropwise with stirring. All microwave reaction times include a 1-minute ramp to the listed temperature. After the reaction was complete, the mixture was allowed to cool for 3–10 min, after which it was treated with excess methanol and then stirred for 10 min at room temperature. The methylphosphonic acid product was isolated as a colorless oil by drying in vacuo to a constant weight. For reactions in sulfolane, the product was not isolated. All experiments were repeated in triplicate to verify reproducibility. Control reactions were also performed, in which all reaction conditions were unchanged except for the replacement of microwave heating with a thermally heated sand bath. All starting diesters (Table 1) were dealkylated to the same product, methylphosphonic acid, producing essentially identical ^1^H and ^31^P NMR spectra, so we reproduce representative product spectra for the methylphosphonic acid product from the microwave BTMS dealkylation of dimethyl methylphosphonate in ACN at 40 °C for 10 m: ^1^H NMR (D_2_O) δ: 1.20–1.24 (d) ppm (Figure S1); ^31^P NMR (D_2_O) δ: 31.05 ppm (Figure S2) These values are consistent with previously reported spectra data: ^31^P NMR (D_2_O) δ: 31.11 and ^1^H NMR (D_2_O) δ: 1.24–1.31 [30]. 

General procedure for the microwave dealkylation of trimethyl- and triethyl phosphonoacetate (Table 2, Entries 1 and 2, respectively), diethyl phosphonoacetate (Table 2, Entry 3), and diethyl (bromodifluoromethyl)phosphonate (Table 2, Entry 4): the procedure was the same as for the methylphosphonate esters; however, in the case of trimethyl- and triethyl phosphonoacetate, the solvent and any excess BTMS were removed under reduced pressure before treatment with methanol, and the reactions were carried out under dry nitrogen as an extra precaution against moisture. This was necessary due to the sensitivity of carboxylate esters to the HBr produced when BTMS is exposed to moisture. The ^31^P NMR spectra of product mixtures in CDCl_3_ were obtained for the bis(trimethylsilyl) esters of (2-methoxy-2-oxoethyl)phosphonic acid (Figure S3) and (2-ethoxy-2-oxoethyl)phosphonic acid (Figure S4), in addition to the ^1^H and ^31^P NMR spectra of the final phosphonic acid products of Table 2 (Figures S5–S11) [34,35,36].

General procedure for the microwave-promoted BTMS dealkylation of dialkyl ANPs (Table 3). PMEDAP(O*i*Pr)_2_ (0.134 mmol), (S)-PMPDAP(OEt)_2_, or PMEA(OiPr)_2_ (0.140 mmol) were dissolved in 1.5 mL of acetonitrile, and 4 or 6 molar equivalents of BTMS was quickly added. All microwave reaction times included a 1-minute ramp to 60 °C (Table 3). After microwave irradiation for 15 or 30 min, the reaction mixture was allowed to cool for 10 min, excess methanol was added, and the mixture was left to stir at room temperature for 10 min. The product was then dried under reduced pressure, dissolved in ammoniated water, and precipitated by adjustment of the pH to 2–2.5 with HBr. The white crystalline product was then filtered and washed with cold water and acetone or ethanol and dried under vacuum at 45 °C to a constant weight. Representative ^31^P and ^1^H NMR spectra are shown in Figures S12–S17) [15].

## 5. Conclusions

Under the microwave conditions described here (MW-BTMS), the conventional BTMS silyldealkylation of methyl, ethyl, and isopropyl phosphonate diesters is greatly accelerated in aprotic solvents having both low and high dialectric constants, including ACN [10], 1,4-dioxane, DMF, sulfolane, and neat BTMS, requiring in general only a few minutes at 40–60 °C to achieve quantitative yields. A large excess of BTMS is unnecessary, and the reactions are conveniently carried out in unsealed vessels at ambient atmospheric pressure. MW-BTMS offers a striking improvement in efficiency over traditional BTMS silyldealkylations and appears to provide significant advantages, including superior chemoselectivity, over the MW-assisted HCl method [15] to prepare phosphonic acids from the corresponding alkyl esters.

## Figures and Tables

**Table 1:** Results for the dealkylation reactions represented in Scheme 1.

Entry	R	Solvent	BTMS (Molar Ratio to Substrate)	Temp. (°C)	Time: MW (min)	% Yield ^a^	Time: Thermal Heating (h)	% Yield (^31^P NMR)
1	Me	ACN	2	40	10	93 ^a^	1	91
2	Me	ACN	2	60	4	97 ^a^	-	-
3	Et	ACN	2	40	15	93 ^a^	7	80
4	Et	ACN	2	60	5	97 ^a^	-	-
5	iPr	ACN	4	40	90	97 ^a^	-	-
6	iPr	ACN	2	60	30	98 ^a^	6	85
7	Me	1,4-Dioxane	2	40	5	>99 ^a^	1	95
8	Me	1,4-Dioxane	2	60	2	>99 ^a^	-	-
9	Me	1,4-Dioxane	2	80	2	>99 ^a^	-	-
10	Et	1,4-Dioxane	2	40	15	95 ^a^	7	74
11	Et	1,4-Dioxane	2	60	6	96 ^a^	-	-
12	Et	1,4-Dioxane	2	80	3	96 ^a^	2	48
13	iPr	1,4-Dioxane	2	80	30	>99 ^a^	4	89
14	Me	Neat BTMS	6	40	2	>99 ^a^	-	-
15	Et	Neat BTMS	6	40	2	98 ^a^	-	-
16	iPr	Neat BTMS	6	40	15	>99 ^a^	-	-
17	Me	DMF	2	40	4	97 ^a^	-	-
18	Me	DMF	2	60	2	94 ^a^	-	-
19	Et	DMF	2	40	6	98 ^a^	-	-
20	Et	DMF	2	60	2	98 ^a^	4	62
21	Et	DMF	2	80	2	95 ^a^	4	98
22	iPr	DMF	2	80	20	96 ^a^	-	-
23	iPr	DMF	2	100	5	97 ^a^	1	73
24	iPr	DMF	2	110	2	95 ^a^	-	-
25	Me	Sulfolane	2	40	4	>99 ^b^	-	-
26	Me	Sulfolane	2	60	2	>99 ^b^	-	-
27	Et	Sulfolane	2	40	15	100 ^b^	-	-
28	Et	Sulfolane	2	60	2	100 ^b^	4	40
29	iPr	Sulfolane	2	60	10	100 ^b^	-	-
30	iPr	Sulfolane	2	80	2	100 ^b^	4	92

^a^ Isolated yield of methylphosphonic acid; yields of bis(trimethylsilyl) methylphosphonate intermediate were >99% by ^31^P NMR. ^b^ Yield of bis(trimethylsilyl) ester by ^31^P NMR.

**Table 2:** Results for microwave BTMS dealkylation reactions on mixed carboxylate–phosphonate esters, as well as on 2-(diethoxyphosphoryl)acetic acid and diethyl(bromodifluoro)phosphonate.

Entry	Substrate	Solvent	BTMS (Molar Ratio to Substrate)	Temp. (°C)	Time: MW (min)	% Yield (NMR ^a^/Isolated ^b^)
1	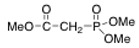	ACN	2	60	10	100/98
2	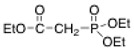	ACN	2	60	15	100/96
3	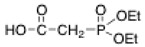	ACN	6	60	30	100/99
4	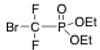	ACN	6	60	60	100/85

^a 31^P and ^1^H NMR yields of bis(trimethylsilyl) phosphonate silyldealkylation products, which were both quantitative. ^b^ Isolated yield of phosphonic acid.

**Table 3:** BTMS dealkylations of acyclic nucleoside phosphonate esters using microwave irradiation.

Entry	Substate	Structure	Solvent	BTMS (Molar Ratio to Substrate)	Temp. (°C)	Time: MW (min)	% Yield	Time: Thermal Heating (h)
1	PMEDAP (OiPr)_2_	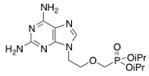	ACN	4	60	30	>99 ^a^	-
			ACN	6	60	15	>99 ^a^/91 ^b^	4
2	(*S*)-PMPDAP (OEt)_2_	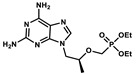	ACN	4	60	15	>99 ^a^/82 ^b^	4
3	PMEA (OiPr)_2_	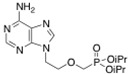	ACN	6	60	15	>99 ^a^/85 ^b^	5

^a 31^P and ^1^H NMR yield of bis(trimethylsilyl) phosphonate silyldealkylation product. ^b^ Isolated yield of phosphonic acid.

## Data Availability

The data presented in this study are available in the article and Supplementary Material.

## Supplementary Materials

The following are available online at https://www.mdpi.com/article/10.3390/molecules28083497/s1: Figures S1–S17: ^31^P and ^1^H NMR spectra of dealkylated products from Table 1, Table 2 and Table 3; Figure S18: depiction of the MW apparatus used in the synthetic experiments.
