# Supplementary material for: Microwave-Accelerated McKenna Synthesis of Phosphonic Acids: An Investigation
Source: Molecules. 2023 Apr 15;28(8):3497. doi: 10.3390/molecules28083497 (PMC10144917; doi:10.3390/molecules28083497)
Supplement: Supplementary file 1 [file molecules-28-03497-s001.zip › molecules-1894944-supplementary.pdf]

Supplementary Material

Microwave-Accelerated McKenna Synthesis of Phosphonic Acids: an Investigation

Dana Mustafa[1,2], Justin M. Overhulse[1], Baoris A. Kashemirov[1] and Charles E. McKenna[1]*

[1] University of Southern California, Department of Chemistry, Los Angeles, CA 90089, USA
[2] Current address: Dentons US LLP, San Diego, CA 92121
***** Correspondence: mckenna@usc.edu

Table of Contents

Figure S1. Representative ^{1}H NMR spectrum of methylphosphonic acid. 2

Figure S2. Representative ^{31}P NMR spectrum of methylphosphonic acid. 3

Figure S3. ^{31}P NMR spectrum of ester of (2-methoxy-2-oxoethyl)phosphonic acid. 4

Figure S4. ^{31}P NMR spectrum of ester of (2-ethoxy-2-oxoethyl)phosphonic acid. 5

Figure S5. ^{1}H NMR spectrum of (2-methoxy-2-oxoethyl)phosphonic acid. 6

Figure S6. ^{31}P NMR spectrum of (2-methoxy-2-oxoethyl)phosphonic acid. 7

Figure S7. ^{1}H NMR spectrum of (2-ethoxy-2-oxoethyl)phosphonic acid. 8

Figure S8. ^{31}P NMR spectrum of (2-ethoxy-2-oxoethyl)phosphonic acid. 9

Figure S9. ^{1}H NMR spectrum of phosphonoacetic acid. 10

Figure S10. ^{31}P NMR spectrum of phosphonoacetic acid. 11

Figure S11. ^{31}P NMR spectrum of (bromodifluoromethyl)phosphonic acid. 12

Figure S12. ^{1}H NMR spectrum of 9-[2-(phosphonomethoxy)ethyl]-2,6-diaminopurine (PMEDAP). 13

Figure S13. ^{31}P NMR spectrum of PMEDAP. 14

Figure S14. ^{1}H NMR spectrum of 9-[(2-(S)-(phosphonomethoxy)propyl-2,6-diaminopurine ((S)-PMPDAP). 15

Figure S15. ^{31}P NMR spectrum of (S)-PMPDAP. 16

Figure S16. ^{1}H NMR spectrum of 9-(2-phosphonomethoxy)ethyladenine (PMEA). 17

Figure S17. ^{31}P NMR spectrum of PMEA. 18

Figure S18. Microwave reactor used in the synthetic experiments. 19

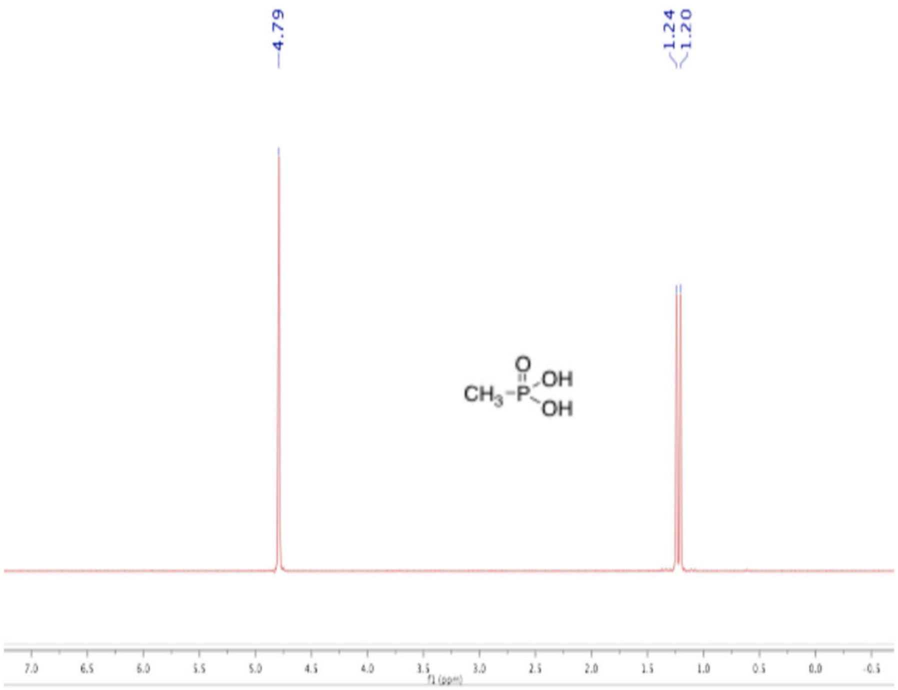

Figure S1. Representative ^{1}H NMR spectrum of methylphosphonic acid.
Product obtained from microwave reactions listed in Table 1. Reaction: Table 1, Entry 1. ^{1}H NMR (500 MHz, D_2O) δ: 1.22 (d).

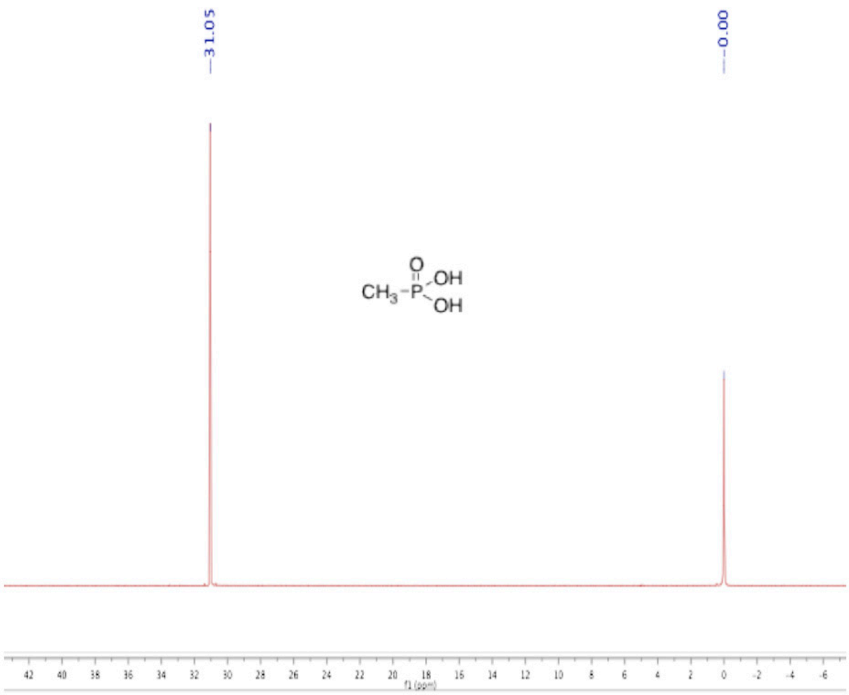

Figure S2. Representative ^{31}P NMR spectrum of methylphosphonic acid.
Product obtained from microwave reactions listed in Table 1. Reaction: Table 1, Entry 1. ^{31}P NMR (202 MHz, D_2O, external H_3PO_4 standard (0 ppm)) δ: 31.05.

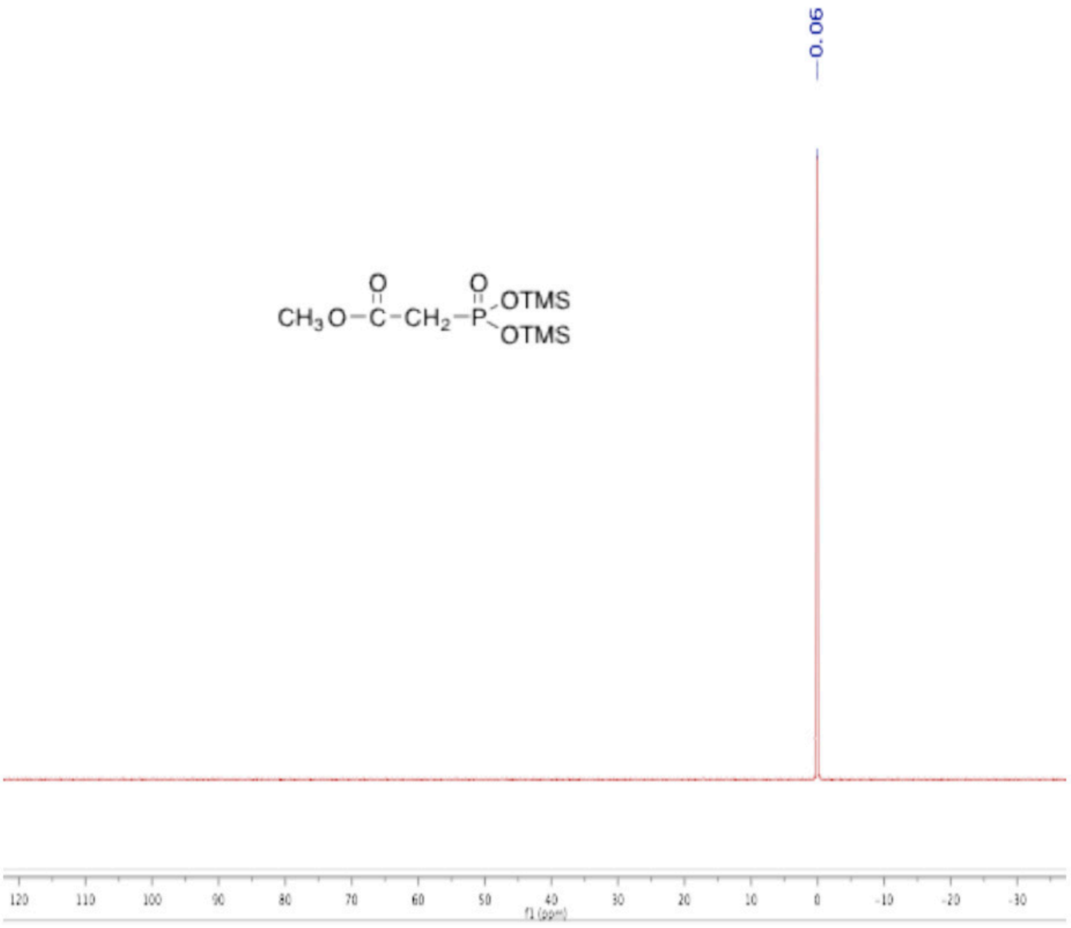

Figure S3. ^{31}P NMR spectrum of ester of (2-methoxy-2-oxoethyl)phosphonic acid.
Product of the BTMS microwave reaction mixture containing the silyl ester of (2-methoxy-2-oxoethyl)phosphonic acid. The single peak demonstrates the selectivity of BTMS for phosphonate esters. Reaction: Table 2, Entry 1. ^{31}P NMR (202 MHz, $CDCl_3$) δ: 0.06.

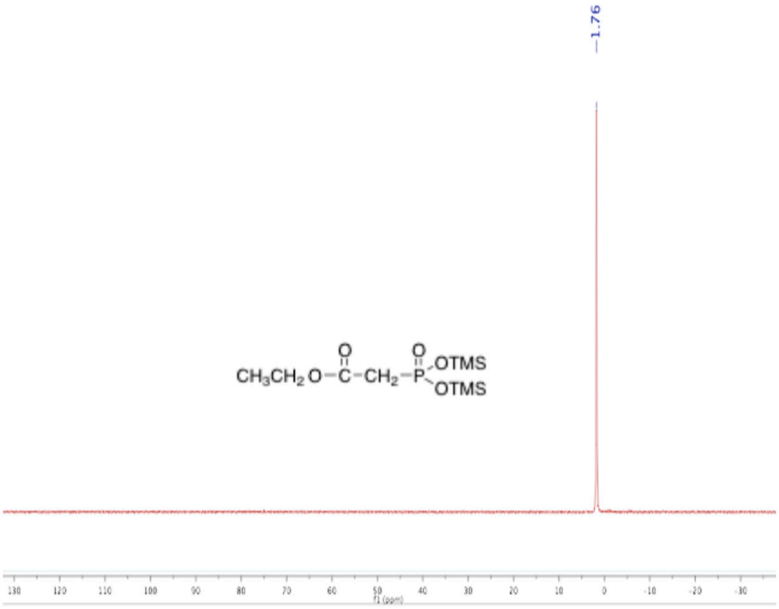

Figure S4. ^{31}P NMR spectrum of ester of (2-ethoxy-2-oxoethyl)phosphonic acid.
Product of the BTMS microwave reaction mixture containing the silyl ester of (2-ethoxy-2-oxoethyl)phosphonic acid. The single peak demonstrates the selectivity of BTMS for phosphonate esters. Reaction: Table 2, Entry 2. ^{31}P NMR (202 MHz, $CDCl_3$) δ: 1.76.

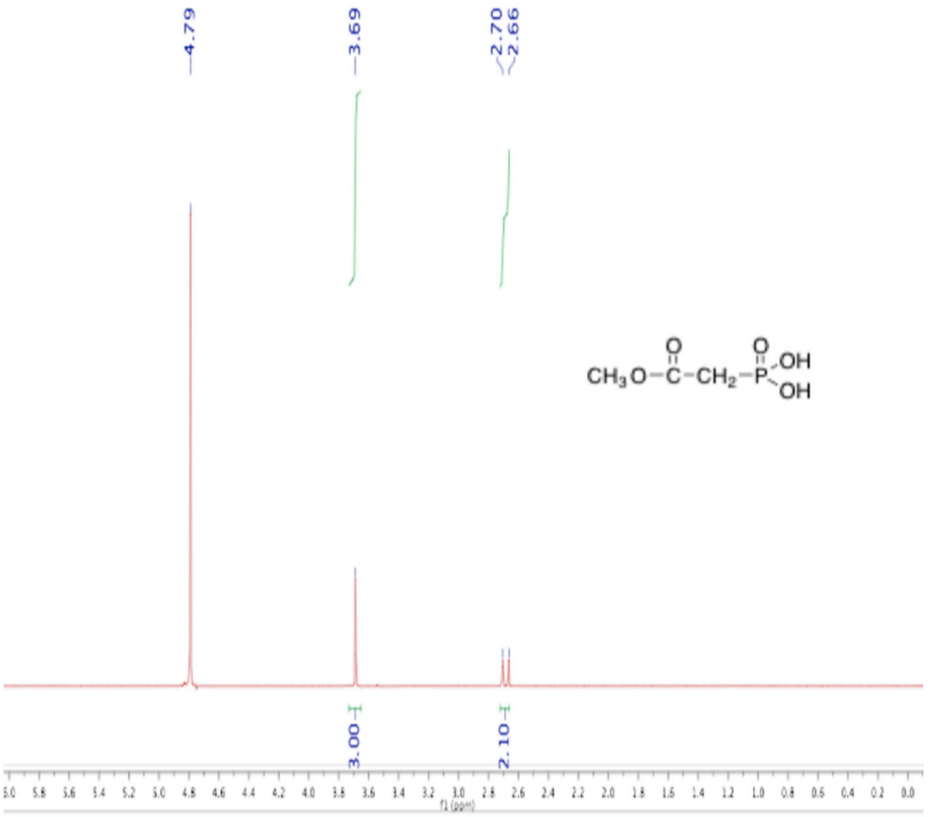

Figure S5. ^{1}H NMR spectrum of (2-methoxy-2-oxoethyl)phosphonic acid.
Product from the BTMS microwave reaction of trimethylphosphonoacetate after desilylation. Reaction: Table 2, Entry 1. ^{1}H NMR (500 MHz, D_2O, pH 7.6) δ: 2.68 (d, 2H), 3.69 (s, 3H).

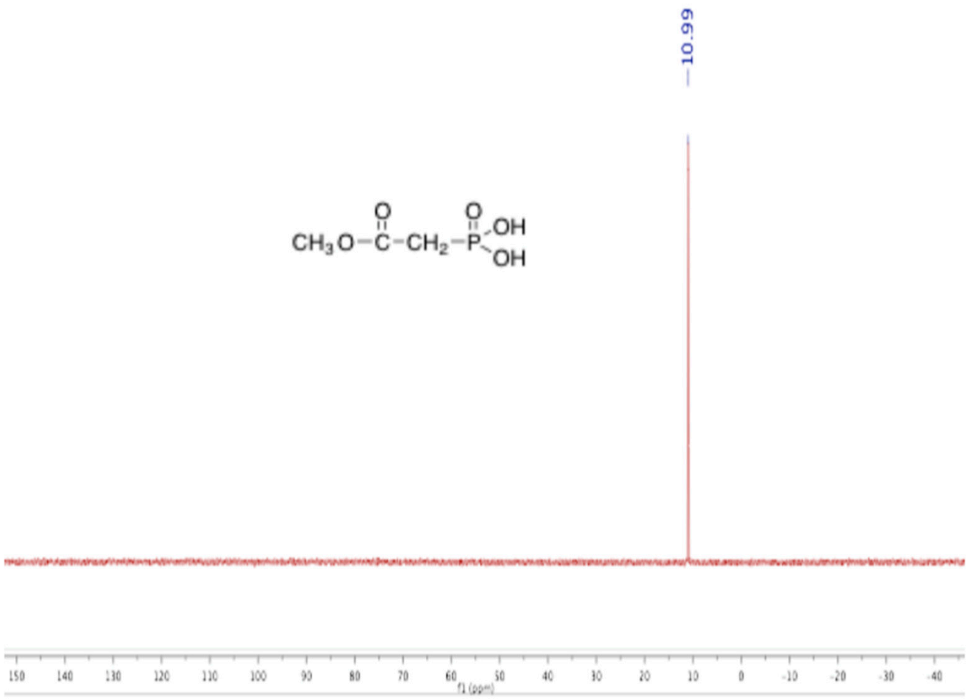

Figure S6. ^{31}P NMR spectrum of (2-methoxy-2-oxoethyl)phosphonic acid.
Product of the BTMS microwave reaction of trimethylphosphonoacetate after desilylation.
Reaction: Table 2, Entry 1. ^{31}P NMR (202 MHz, D_2O, pH 7.6) δ: 10.99.

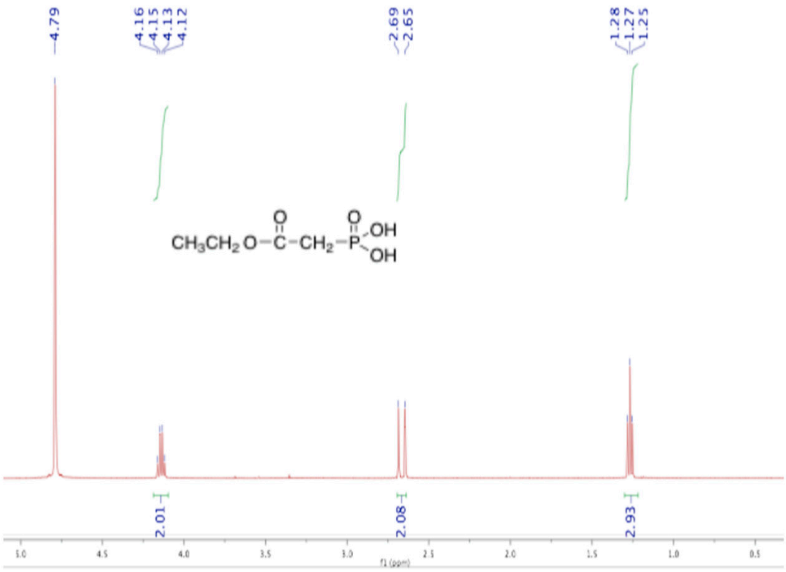

Figure S7. ^{1}H NMR spectrum of (2-ethoxy-2-oxoethyl)phosphonic acid.

Product of the BTMS microwave reaction of triethylphosphonoacetate after desilylation. Reaction: Table 2, Entry 2. ^{1}H NMR (500 MHz, D_2O, pH 8.2) δ: 1.27 (t, 3H), 2.67 (d, 2H), 4.14 (q, 2H).

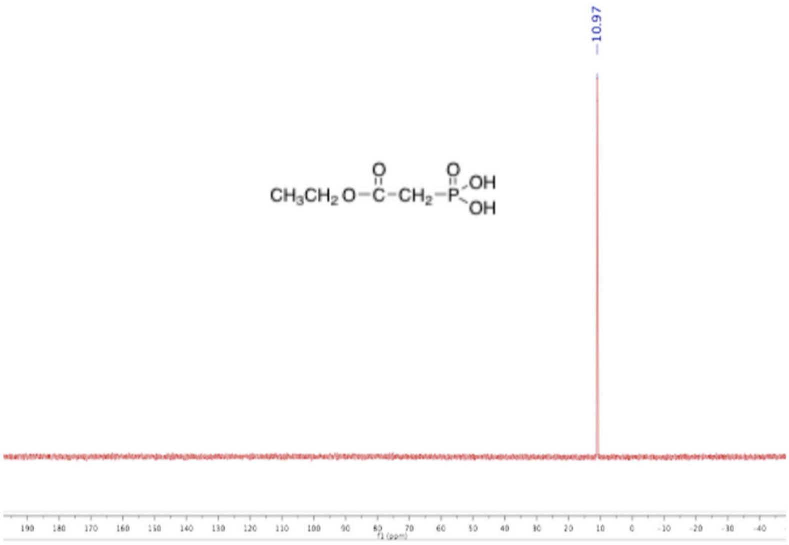

Figure S8. ^{31}P NMR spectrum of (2-ethoxy-2-oxoethyl)phosphonic acid.
Product of the BTMS microwave reaction of triethylphosphonoacetate after desilylation. Reaction: Table 2, Entry 2. ^{31}P NMR (202 MHz, D_2O, pH 8.2) δ: 10.97.

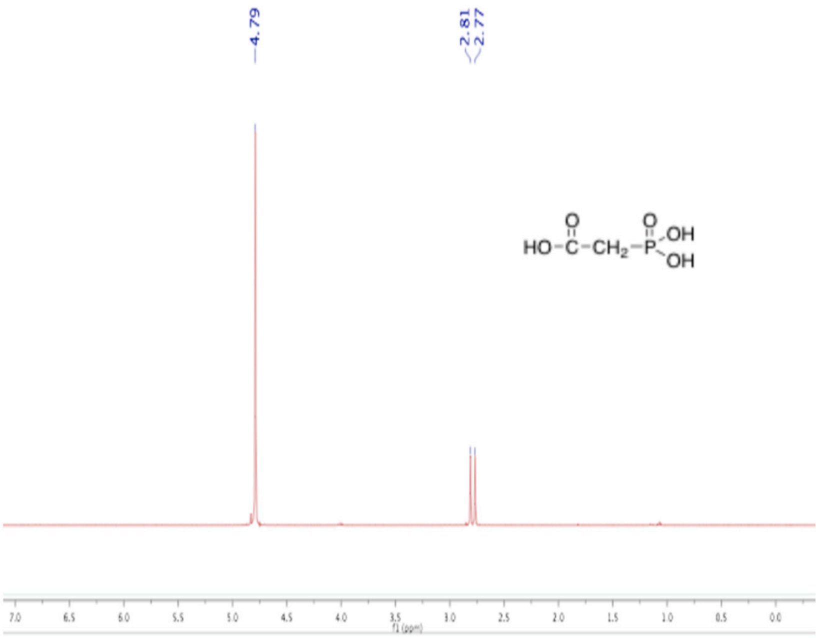

Figure S9. ^{1}H NMR spectrum of phosphonoacetic acid.
Product of the BTMS microwave reaction of 2-(diethoxyphosphoryl)acetic acid after desilylation. Reaction: Table 2, Entry 3. ^{1}H NMR (500 MHz, D_2O) δ: 2.79 (d).

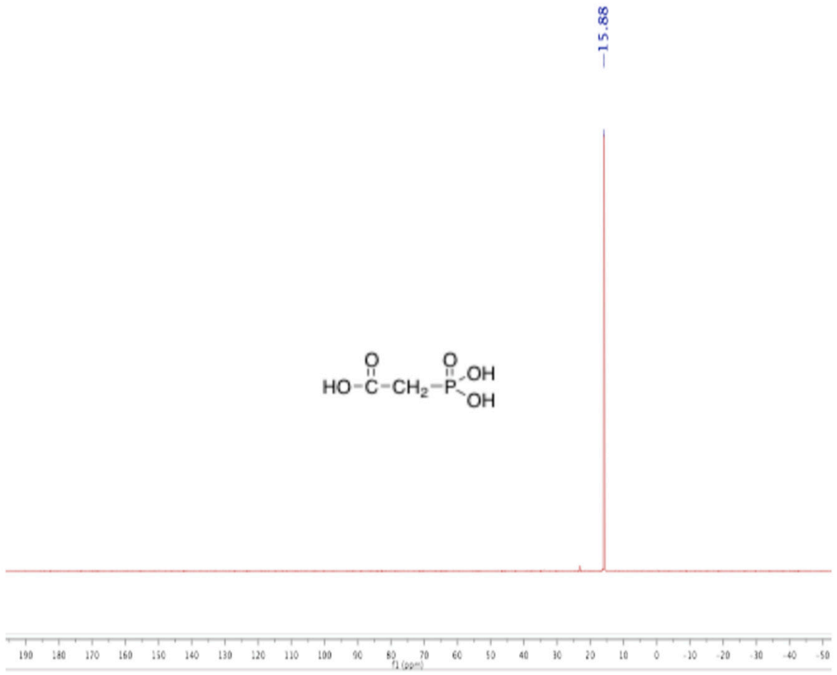

Figure S10. ^{31}P NMR spectrum of phosphonoacetic acid.
Product of the BTMS microwave reaction of 2-(diethoxyphosphoryl)acetic acid after desilylation.
Reaction: Table 2, Entry 3. ^{31}P NMR (202 MHz, D_2O) δ: 15.88.

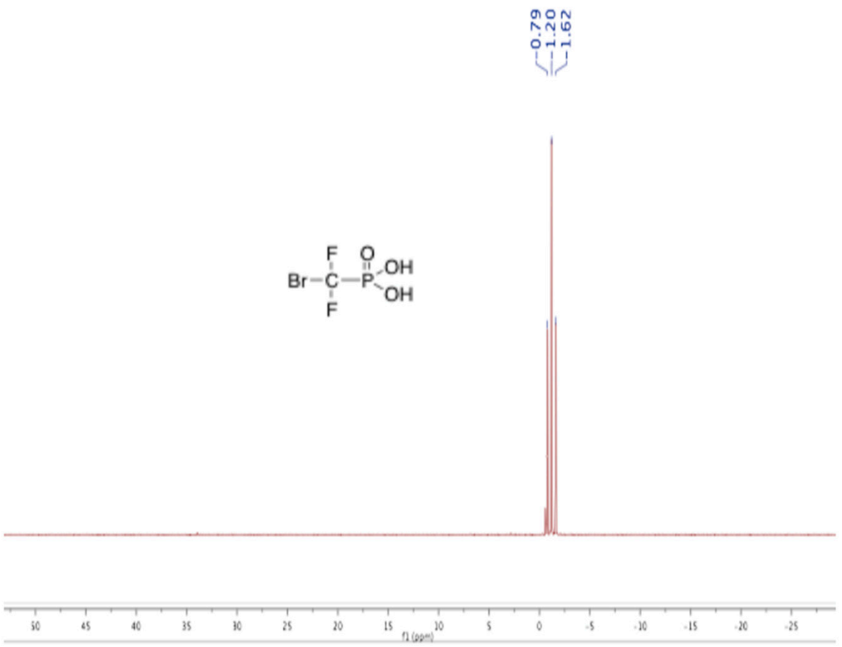

Figure S11. ^{31}P NMR spectrum of (bromodifluoromethyl)phosphonic acid.
Product of the BTMS microwave reaction of diethyl (bromodifluoromethyl)phosphonate after desilylation. Reaction: Table 2, Entry 4. ^{31}P NMR (202 MHz, D_2O) δ: -1.20 (t).

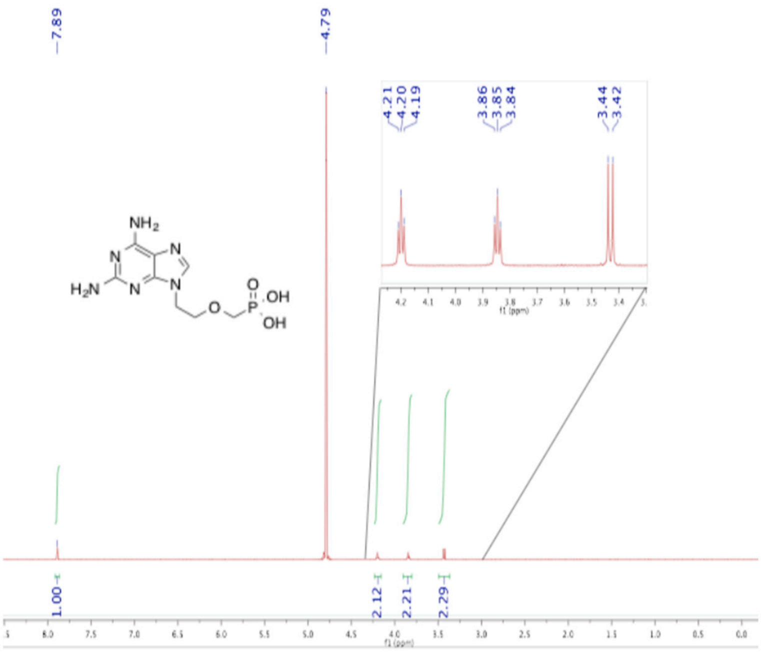

Figure S12. ^{1}H NMR spectrum of 9-[2-(phosphonomethoxy)ethyl]-2,6-diaminopurine (PMEDAP). Product of the BTMS microwave reaction of PMEDAP(OiPr)$_2$ after desilylation. Reaction: Table 3, Entry 1. ^{1}H NMR (500 MHz, D_2O) δ: 3.43 (d, 2H), 3.85 (t, 2H), 4.20 (t, 2H), 7.89 (s, 1H).

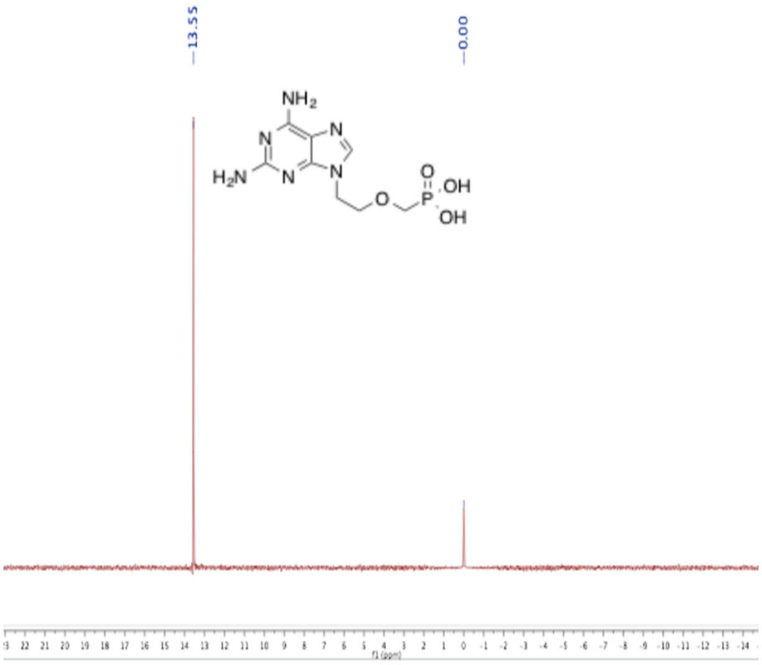

Figure S13. ^{31}P NMR spectrum of PMEDAP.
Product of the BTMS microwave reaction of PMEDAP(OiPr)$_2$ after desilylation. Reaction: Table 3, Entry 1. ^{31}P NMR (202 MHz, D_2O, external H_3PO_4 standard (0 ppm)) δ: 13.55.

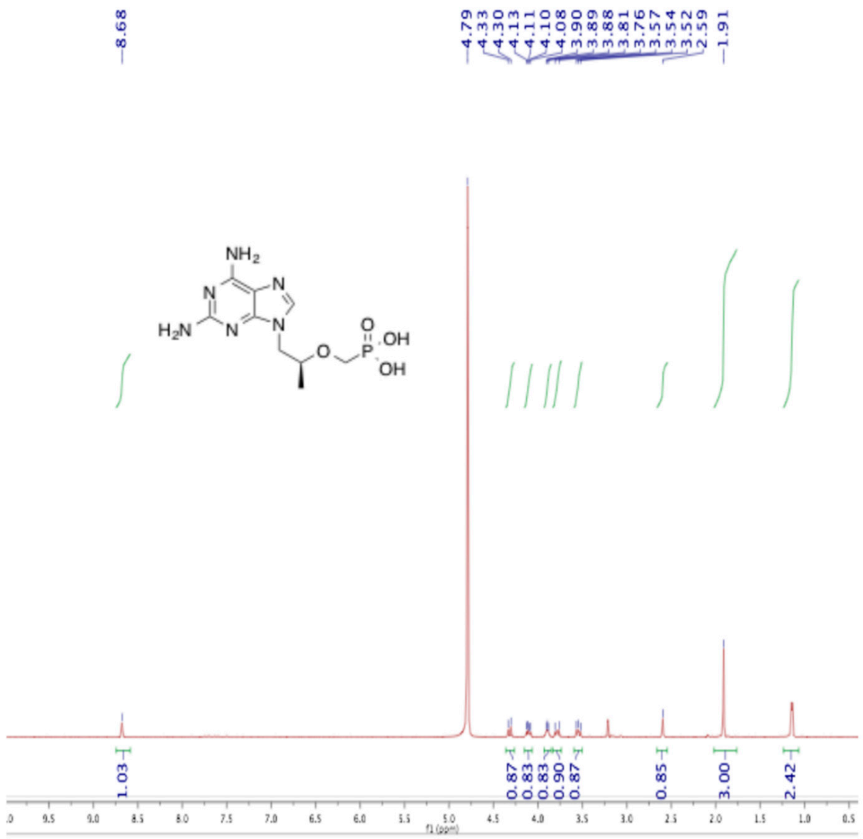

Figure S14. ^{1}H NMR spectrum of 9-[(2-(S)-(phosphonomethoxy)propyl-2,6-diaminopurine ((S)-PMPDAP).
Product of the BTMS microwave reaction of (S)-PMPDAP(OEt)$_2$ after desilylation. Reaction: Table 3, Entry 2. ^{1}H NMR (500 MHz, D_2O) δ: 1.91 (s, 3H), 2.59 (s, 1H), 3.54 (t, 1H), 3.78 (m, 1H), 3.89 (m, 1H), 4.10 (m, 1H), 4.31 (d, 1H), 8.68 (s, 1H).

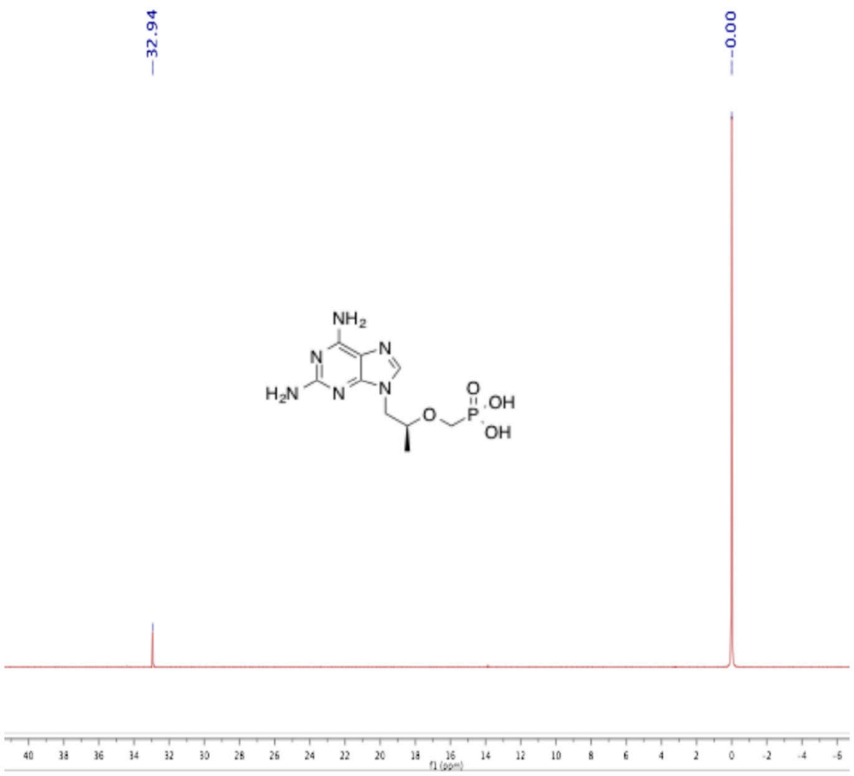

Figure S15. ^{31}P NMR spectrum of (S)-PMPDAP.
Product of the BTMS microwave reaction of (S)-PMPDAP(OEt)$_2$ after desilylation. Reaction: Table 3, Entry 2. ^{31}P NMR (202 MHz, D_2O, external H_3PO_4 standard (0 ppm)) δ: 32.94.

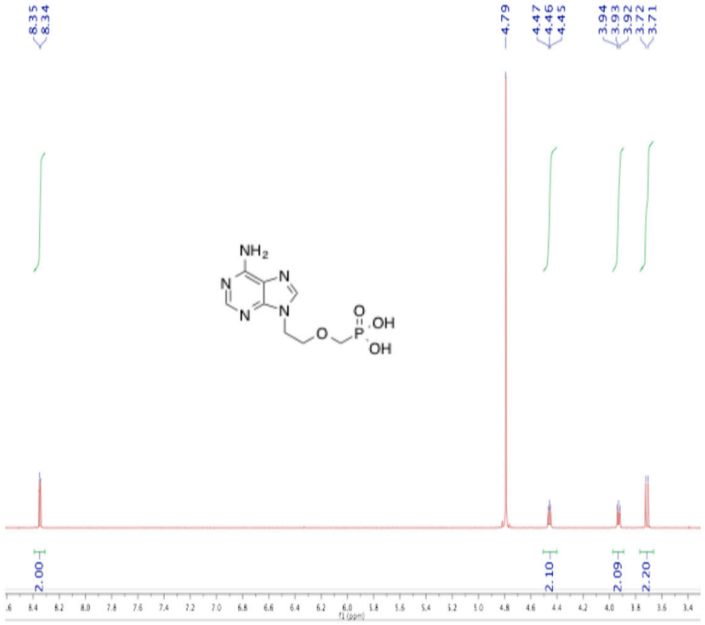

Figure S16. ^1H NMR spectrum of 9-(2-phosphonomethoxy)ethyladenine (PMEA).
Product of the BTMS microwave reaction of PMEA(OiPr)$_2$ after desilylation. Reaction: Table 3, Entry 3. ^1H NMR of (500 MHz, D_2O) δ: 3.71 (d, 2H), 3.93 (t, 2H), 4.46 (t, 2H), 8.34 (d, 2H).

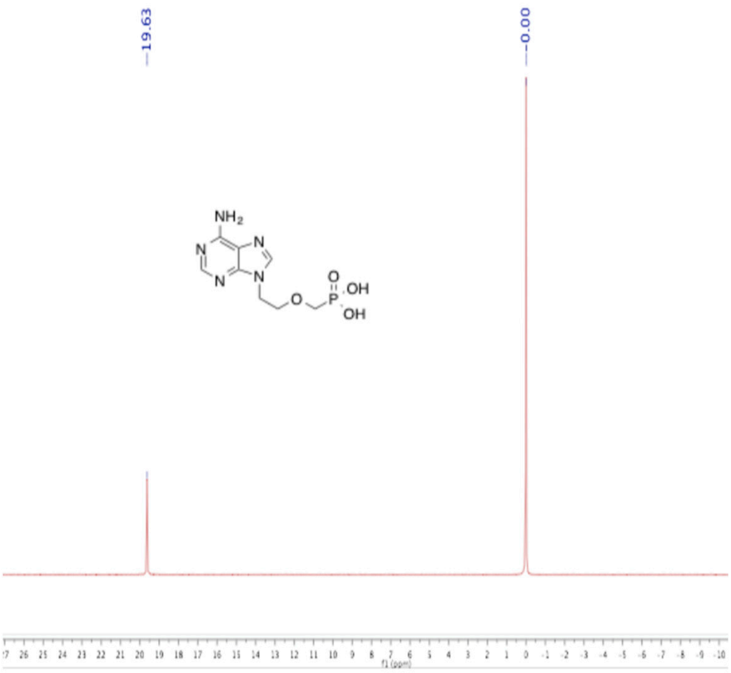

Figure S17. ^{31}P NMR spectrum of PMEA.
Product of the BTMS microwave reaction of $PMEA(OiPr)_2$ after desilylation. Reaction: Table 3, Entry 3. ^{31}P NMR (202 MHz, D_2O, external H_3PO_4 standard (0 ppm)) δ: 19.63.

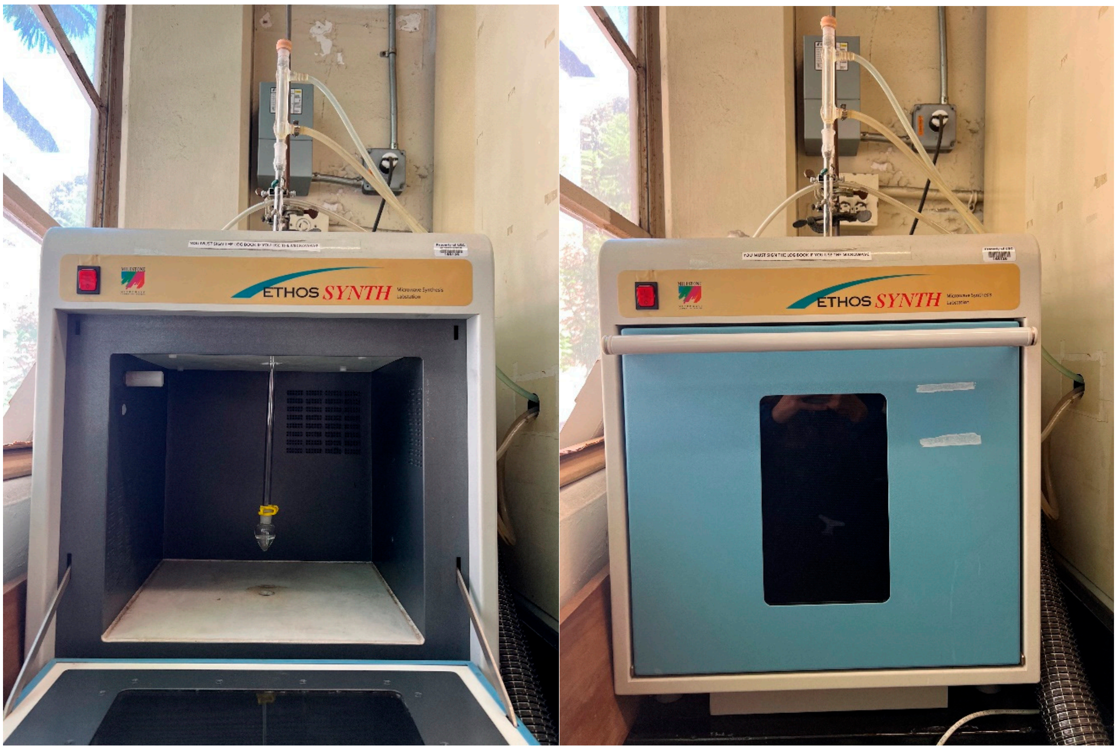

Figure S18. Microwave reactor used in the synthetic experiments.
The left panel shows the reaction flask attached to the built-in condenser of the Ethos SYNTH reactor. The right panel shows the setup with the door shut, during reaction.
